# 

**Published:** 1864-06

**Authors:** 


### CONTENTS.

### ORIGINAL CONTRIBUTIONS.

ART. XXIII. REPORT OF COMMITTEE ON PRACTICAL MEDICINE. By N. S. DAVIS, M.D., Prof. of Practical and Clinical Medicine, .......... 321

ART. XXIV. CASE OF HYDROPHOBIA. By R. S. ADDISON, M.D., of Chicago, .......... 345

### SELECTIONS.

Of Heart-Clot as a Cause of Death in Diphtheria. By J. Forsyth Meigs, M.D., Fellow of the College of Physicians of Philadelphia, one of the Physicians to the Pennsylvania Hospital, .......... 349

Oxygennesis for the Instantaneous Production of Pure Oxygen without Heat. By Mr. J. Robbins, .......... 362

### PROCEEDINGS OF SOCIETIES.

Proceedings of the Twelfth Annual Meeting, Fourteenth Anniversary, of the Illinois State Medical Society. Held in Chicago, May 3d, 4th, and 5th, 1864, .......... 368

### EDITORIAL.

Illinois State Medical Society, .......... 379

Cleft Palate, Congenital and Otherwise. Their Remedies. By E. A. BOGUE, D.D.S., .......... 379

Obstinate Constipation, .......... 381

